# A Novel Peptide Thrombopoietin Mimetic Designing and Optimization Using Computational Approach

**DOI:** 10.3389/fbioe.2016.00069

**Published:** 2016-08-31

**Authors:** Vimal Kishor Singh, Neeraj Kumar, Manisha Kalsan, Abhishek Saini, Ramesh Chandra

**Affiliations:** ^1^Founder and O/I Stem Cell Research Laboratory, Department of Biotechnology, INSPIRE Faculty, Delhi Technological University, Delhi, India; ^2^Department of Chemistry, University of Delhi, Delhi, India; ^3^Stem Cell Research Laboratory, Department of Biotechnology, Delhi Technological University, Delhi, India

**Keywords:** thrombopoietin, thrombopoietin receptor, mimetic library designing, molecular docking, molecular dynamics simulation

## Abstract

Thrombopoietin receptor (TPOR) is a cytokine receptor protein present on the cell surface. The activation of TPOR by thrombopoietin (TPO) (a glycoprotein hormone) triggers an intracellular cascade of megakaryocytopoiesis for the formation of platelets. Recent studies on *ex vivo* megakaryocytopoiesis have evolved the possibilities of therapeutics uses. These findings have paved the way for the development of various TPO alternatives (recombinant TPO, peptide, and non-peptide TPO mimetics), which are useful in regenerative medicine. However, these alternatives possess various limitations such as induction of autoimmune effects, high production cost, low specificity, and hence activity. In the present study, a novel peptidic TPO mimetic was designed through computational studies by studying the binding sites of TPO and TMP to TPOR and analogs of known mimetics. Screening of combinatorial library was done through molecular docking using ClusPro. These studies indicated mimetic-9 as a significant molecule since it was found to have better binding score of −938.8 kcal/mol with seven hydrogen bonds and a high number of hydrophobic interactions, than known mimetic TMP with docking score of −798.4 kcal/mol and TMP dimer with docking score of −811.9 kcal/mol for TPOR. Mimetic9-TPOR complex was further assessed by the molecular dynamics simulation, and their complex was found to be stable with an RMSD value of 0.091 Å. While studying the parameters, mimetic-9 was found to have overall good physiochemical properties with positive grand average hydropathy (GRAVY) score and high instability index score and was found to be localized in the extracellular region. The designed mimetic-9 might prove to be a useful lead molecule for mimicking the role of TPO for *in vitro* platelet production with higher efficiency.

## Introduction

Blood is a vital connective tissue responsible for the transport of gases and metabolites in vertebrates. It consists of cellular and acellular components, such as erythrocytes (red blood cells), leukocytes (white blood cells), platelets (thrombocytes) (cellular components), and plasma (acellular component), including various types of solubilized/suspended proteins, growth factors/cytokines/hormones, vitamins, minerals, and water. One of the important cellular component i.e., platelets are majorly responsible for wound healing and inhibition of the bleeding. These cells stick to the locations of tissue damage and recruit other factors that aid hemostasis and healing (Kuter, 1991). During homeostasis, platelets concentration in human blood is approximately 50 × 10^9^ cells/L, and a decline in the platelets concentration for any reason (< 10 × 10^9^ cells/L) is considered as inadequate hemostasis that needs an immediate supportive measure for survival (Beutler, 1993). There are many conditions, such as thrombocytosis, thrombocytopenia, and dengue fever, which result in decreased platelet counts due to the destruction of platelets and bone marrow megakaryocytes (MKs) caused by autoantibodies and insufficient thrombopoietin (TPO) levels. Apart from that, there are many syndromes, such as gray platelet syndrome, platelet alpha-granule deficiency, which are associated with the dysfunctioning of platelets and not their concentration as such.

The adequate concentration of platelets (and other cellular components) is maintained by a tightly regulated hierarchy of various progenitors/precursors and their interactions with corresponding growth factors/cytokines *in vivo* (Matsumura and Kanakura, 2002). The hematopoietic stem cells (HSCs) differentiate into different progenitors, including the megakaryocyte-erythroid progenitor (MEP) and the common myeloid progenitor (CMP), committed for different cell lineages. MEPs give rise to erythroid and megakaryocytic lineages, which produce erythrocytes and MKs, respectively (Woolthuis and Park, 2016). MKs contain a much higher concentration of DNA (64 times to the average concentration; 128 N). The immature MKs form mature MKs, which generates platelets.

In thrombocytopenia, a decrease in platelet counts elicits the increased levels of free TPO to restore the number and size of MKs leading to the increased production of platelets. The polyploidy of these MKs increases from 16 N to 32 N, and the production of platelets increases to up to 11-fold (Harker, 1968; Jackson et al., 1984; Kuter and Rosenberg, 1990). This effect is carried out by TPO (Kelemen et al., 1958).

Thrombopoietin (also called *c*-MPL ligand) is a cytokine having a crucial role in proliferation and maturation of MKs. The primary site for the synthesis of TPO’s mRNA and protein is liver, while in small amounts also produced in kidney, testes, and brain (Shimada et al., 1995; Stoffel et al., 1996). TPO is released immediately, once synthesized. Once TPO binds to its thrombopoietin receptor (TPOR) (*c*-MPL), a cascade of signaling pathways is induced, which in co-operation with transcription factors [such as signal transducer and activator of transcription 3 (STAT3) and STAT5] induce the proliferation and maturation of MKs (Drachman and Kaushansky, 1997).

Considering such an important role in the maintenance of platelet counts, various measures have been developed by different research groups in the treatment of thrombocytopenia and the related disorders. First generation agents, including recombinant TPO, were studied under various extensive clinical studies. However, use of recombinant TPO molecule was reported to induce auto immune antibodies due to structural similarities with endogenous TPO, and eventually, the production of these molecules was stopped in 1998 (Bartley et al., 1994; de Sauvage et al., 1994; Kuter et al., 1994; Lok et al., 1994; Kato et al., 1995; Kuter and Begley, 2002).

Second generation agents included recombinant TPO fusion proteins [e.g., Promegapoietin (TPO/IL3 fusion protein)], TPO peptide mimetics (e.g., Fab59, AMG531, and PEG-TPOmp), non-peptide TPO mimetics (e.g., Eltrombopag, AKR-501), and TPO agonist antibodies (e.g., minibodies and domain subclass converted agonist antibodies). Cwirla et al. (1997) identified a 14 amino acid peptide with no sequence homology to TPO and can efficiently bind to TPOR. Further studies have shown that dimerization of this peptide might increase its biological activity. However, these mimetics had a short circulating half-life. Frederickson et al. (2006) developed Fab59 by inserting the first peptide of this dimer into complementarity determining region 3 (CDR3) of the heavy chain and the second peptide into CDR2 of the light chain of human Fab. The increase in growth of TPO-dependent cell lines was equivalent to that of TPO, but the increase in platelet counts was 30-fold less. Peg-TPOmp was found to induce a dose-dependent rise in the levels of endogenous TPO. AMG531 or romiplostim or *N*-plate was the first TPOR agonist, which entered clinical trials; binds to TPOR in a manner similar to TPO and is found to increase platelet counts in both splenectomized and non-splenectomized patients. Romiplostim has been found to cause bone marrow fibrosis in a dose-dependent manner, which decreases if the therapy is discontinued. In patients with thrombocytopenia associated with myelodysplastic syndromes, around one-third of the patients experienced increased reticulin fibrosis (Teofili et al., 2010).

There is another approach to the second generation agents that includes non-peptide mimetics. For example, eltrombopag is a non-peptide mimetic (hydrazone small molecule), which is administered orally and binds to TPOR in its transmembrane region to activate the pathway (Erickson-Miller et al., 2005). Single doses have no effect, but doses in the manner 5, 10, 20, 30, 40 up to 10 days have been shown to increase platelet counts in a dose-dependent manner for doses above 30 mg. The oral absorption of eltrombopag has been found to be affected by some foods and medications. Its studies indicate no development of bone marrow fibrosis for up to 44 weeks, but data indicate that subset of patients become prone to bone marrow fibrosis when TPOR is stimulated. Small molecules e.g., AKR-501 or YM477 has been found to stimulate the growth of TPO-dependent cell lines, increase the generation of MKs from CD34 + cells (Suzuki et al., 2005). Non-peptide mimetics are known to have some characteristics – they activate the TPOR in a different manner than TPO, are highly species specific, and possess additive effect to TPO.

The third approach is the use of monoclonal antibodies as these are having the long circulating half-life, are well tolerated and non-immunogenic. Minibodies or VB22B sc(Fv)2 is small bivalent antibody fragments, having a size similar to a Fab fragment, prepared by the genetic modification of variable regions of heavy and light chains. It binds and activates the TPOR and increases platelet counts (Orita et al., 2005).

In our study, a novel TPO mimetic peptide was designed using computational methods. Binding pockets of TPO and reported mimetics are important hence a library of mimetics was created and analyzed using the bioinformatics tools. The 3D structure of extracellular domain (ECD) region of TPOR was predicted to study its binding potency with the peptides of the library generated, to shortlist the potent TPO mimetic. Furthermore, the stability of TPO mimetic-TPOR complex was assessed by molecular dynamic simulations along with the analysis of physicochemical properties of the lead mimetic.

## Materials and Methods

### 3D Structure Prediction and Assessment of TPOR

Thrombopoietin receptor binds to TPO from its ECD, so the amino acid sequence of ECD region was retrieved from Uniprot database and searched for 3D structure in protein data bank. In the absence of crystal structure for mammalian TPOR, the 3D structure was generated. Amino acid sequence of TPOR was searched for the identification of suitable template through Basic Local Alignment Search Tool (BLAST) (Camacho et al., 2008).^1^ Due to unavailability of a template with higher query coverage and with good identity, 3D structure was generated through *de novo* method using Iterative Threading ASSEmbly Refinement (I-TASSER).^2^ I-TASSER is web-based protein 3D structure prediction tool based on threading approach. It simulates the generated structure and defines its *C*-score, TM-score, and active sites. *C*-score refers to a confidence level of predicted structure of a given protein sequences where a high *C*-score (range from −5 to 2) indicates an absolute precise quality of the predicted structure. TM-score is structural assessment parameter in which, a smaller distance between the structures is weighted high. TM-score defines the topology of the structure, and it shows that a score more than 0.5 ensures a model of absolute topology (Yang et al., 2015).

Generated TPOR 3D structure was further evaluated by Swiss model workspace.^3^ Swiss server analyzes the various properties viz. solvation energy, torsion angle energy, solvent accessibility, and atom pairwise energy. Estimate of nativeness of these structural properties of the model with comparable features with high-resolution X-ray 3D protein structures is defined by the QMEAN score. Overall quality of the 3D structure was defined by the QMEAN *Z*-score (Benkert et al., 2008). Furthermore, TPOR structure was evaluated for its stereochemical properties through Ramachandran plot. Rampage server^4^ was used to determine the dihedral angles [phi (Φ) and psi (ψ)], and the number of residues lying in favored, allowed, and outlier regions of the protein structure. It also defines the quality of the structure, planarity of the peptide bond, the main chain hydrogen bond energy; Cα chiralities and non-bonded interactions of structure (Lovell et al., 2003).

### Mimetic Library Generation

A library was formed on the basis of the interaction sites of TPO-TPOR, known mimetic TMP-TPOR, and by designing analogs of known TPO mimetics through the literature survey. Library was *in silico* matured by changing amino acids at different positions with hydrophobic amino acids and forming various combinations of predicted mimetics. Hydrophobic amino acids were added to the mimetics since they are known to play an important role in TPOR binding and its activation.

### Receptor Protein Preparation

Thrombopoietin receptor 3D structure derived through *de novo* method was prepared for molecular docking analysis using the protein preparation wizard (PrepWiz) in the Schrödinger software program (Friesner et al., 2006). Water molecules, unnecessary ligand, unwanted bound ligand, and duplication of any chain were removed, and hydrogen atoms were added to the TPOR protein structure, and then structure is selected for energy minimization and followed by optimization for molecular docking analysis.

### Ligands 3D Structure Prediction

The 3D structures of mimetics in the library were predicted by Pepfold3, a peptide structure prediction server.^5^ Pepfold3 is a new 3D structure generation engine. It predicts the structure of peptides based on the Hidden Markov model conformation sampling approach (Shen et al., 2014). All mimetics were imported into the workspace of Pepfold3, and the project name was saved and allowed to run. Predicted 3D structures were saved in PDB format.

### Molecular Docking Analyses of Mimetic Peptide Library with TPOR and Their Evaluation

Interaction analyses of mimetics with TPOR were done using ClusPro protein–protein molecular docking program. ClusPro server is a rigid docking program based on fast Fourier transformation to generate low energy interaction conformations of a protein–protein complex using the pairwise docking potentials. ClusPro clusters the interaction complexes with low energy and analyzes with semi-definite programming based underestimation program, which identifies the stability of the interaction clusters using the medium-range optimization algorithm. Stable interaction complex is refined using Monte–Carlo simulations (Kozakov et al., 2010). Resulting top docking score mimetic was selected, and its docked complex was analyzed to determine the molecular interactions using the Dimplot program of Ligplot+ (v.1.4.5) module. Dimplot determines the involved molecular interactions including the hydrogen bonds and hydrophobic interactions, between receptor and ligand interacting complex (Wallace et al., 1996).

### Molecular Dynamics Simulation of Mimetic-TPOR Complex

Dynamics and stability of the complex between TPOR and shortlisted mimetic were evaluated through the molecular dynamics simulations using MDWeb program. MDWeb is a workspace, which provides standard protocols for molecular dynamics simulations and to scrutinize the interactions trajectories (Hospital et al., 2012). The optimized structure of the complex of TPOR and the mimetic shortlisted from designed mimetic library on the basis of molecular docking was used as input data for molecular simulations. NAMD full MD setup was used for carrying out the simulation. The whole process of simulation includes cleaning of structure, fixing of side chains, addition of hydrogen atoms, neutralization, addition of solvent box, and minimization and equilibration of the system to finally achieve the structure prepared by simulation. The equilibration of the system includes heating the solvent to 300 K and reducing the restraints to just protein backbone. These steps are carried out to provide the structure with the necessary conditions to study its dynamics along a span of time. Once the structure is prepared, the water molecules in it are removed to reduce the size of the system to achieve the dry trajectory for plotting various graphs for the analysis of the simulated complex structure. The structural stability, flexibility, and deviations per residues were assessed by the RMSD value.

### Physicochemical Properties and Biological Activity Assessment of Lead Mimetic

Lead mimetic was analyzed for its physicochemical properties and compared with the TMP. Physicochemical properties of mimetic-9 and TMP including molecular formula, molecular weight, theoretical isoelectric point (pI), the number of negative and positive residues, extinction coefficient, aliphatic index, instability index, and grand average hydropathy (GRAVY) were determined using the Protparam server (Gasteiger et al., 2005).^6^

Molecular weight, extinction coefficient, net charge, and other properties are required to identify the chemical nature of the peptide and evaluate the binding efficiency of mimetic to a specific receptor. Binding efficiency of mimetic is assessed by potency efficiency index, which is defined by percent inhibition at a given compound concentration divided by its molecular weight. The extinction coefficient illustrates absorbance of light by a protein at a certain wavelength and is used to quantify the formation and concentration of peptides during *in vitro* analyses. Instability index value denotes whether the peptide would be stable *in vitro* or not. The instability index estimates the stability of peptides from statistical analysis of experimentally defined stable and unstable proteins. Aliphatic index predicts the total volume occupied by aliphatic side chains and indicates the thermostability of protein. Another important parameter that implies the stability of the protein is GRAVY value. It determines hydrophobicity and hydrophilicity of a protein by adding the hydropathy values of each amino acid residue and dividing by the number of amino acids in the protein sequence. A positive value of GRAVY indicates higher hydrophobicity of proteins. Furthermore, mimetic subcellular localization was determined by using the Cello prediction server v.2.5.^7^ Cello server predicts the localization in humans, based on the multiple *n*-peptide compositions through support vector machine (Yu et al., 2006).

## Results

### 3D Structure Prediction and Assessment of TPOR

Thrombopoietin receptor (human origin) extracellular region amino acids sequence of size 466 amino acids was retrieved from Uniprot database (Uniprot accession number Uniprotkb-P40238). TPOR 3D structure was predicted using I-TASSER. It generated five models of TPOR, which were analyzed for their properties. Among these models, the model-1 was shortlisted with high *C*-score (−1.03), TM-score (0.58 ± 0.14), and RMSD value of 9.5 ± 4.6 Å. *C* and TM-score indicated the TPOR structure to be of good quality with high confidence, and RMSD value showed it has optimal stability and flexibility.

**Figure 1:**
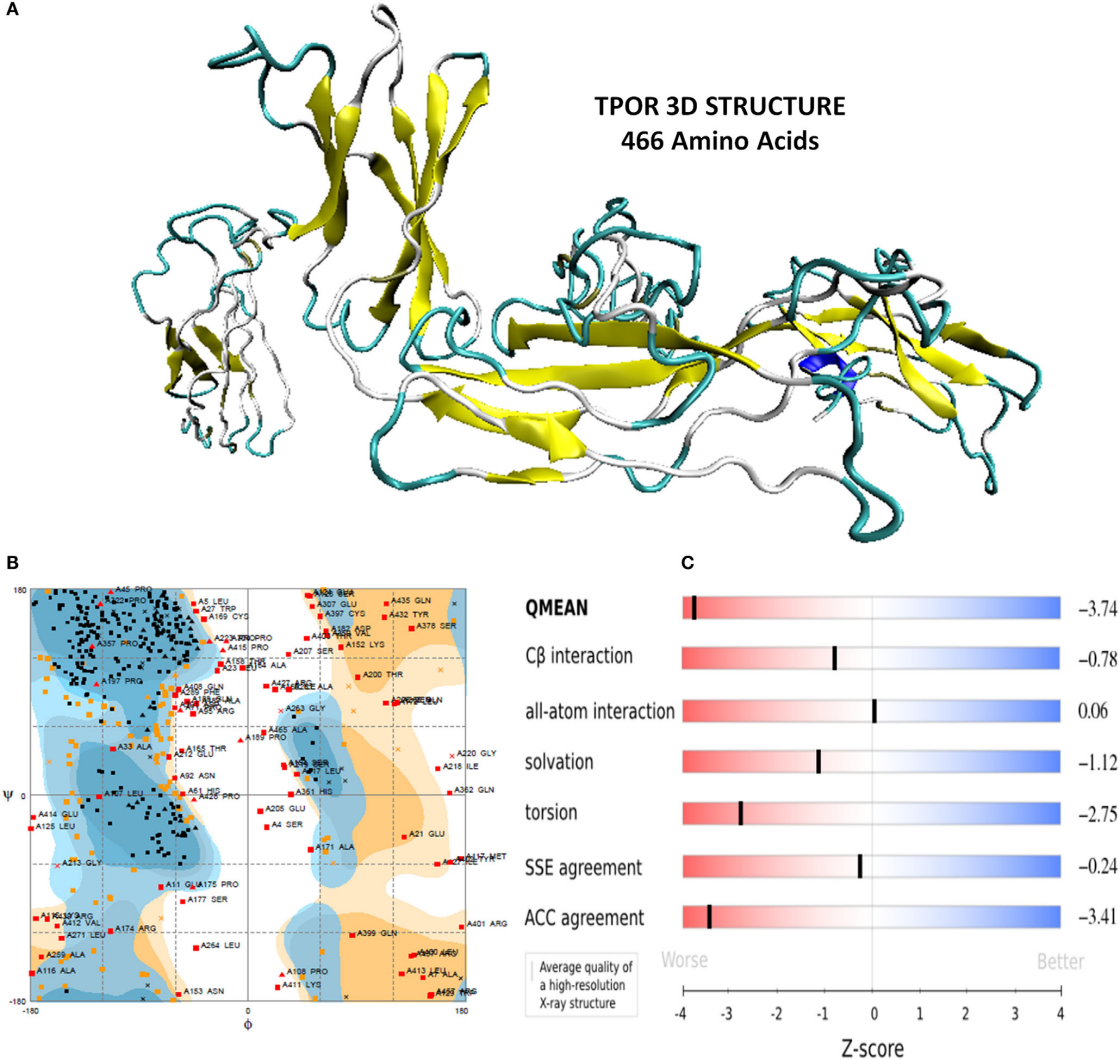
**(A)** Energy minimized structure of TPOR extracellular domain region through I-TASSER **(B)** Ramachandran plot of the 3D structure of TPOR showing the favored and allowed region (81% of residues) **(C)** 3D structural assessment using the Swiss server showed good results with QMEAN score 0.362, for solvation energy, torsion angle energy, solvent accessibility, and atom pairwise energy. The plot depicts the sliders in the light red to blue region, which suggests a good structural quality of TPOR.

Furthermore, TPOR structure was evaluated for its stereochemical and physiochemical properties. Its stereochemical properties were analyzed using Rampage. It showed 60.1% residues of the TPOR structure were in favored region, and 20.9% residues were in the allowed region, and 19% residues were in outlier region. Physiochemical properties were determined by Swiss server and found QMEAN score is 0.362, representing the overall absolute quality of the TPOR structure for the various properties (solvation energy, torsion angle energy, solvent accessibility, and atom pair wise energy). Values for these properties lie in the light red to red region of QMEAN score plot of TPOR structure and indicate good values of physiochemical properties (Figure 1).

### Interaction Analyses of TMP and Mimetic Library with TPOR

The mimetic library was designed on the basis of binding sites of TPO to TPOR, TMP binding sites to TPOR, analogs of reported mimetics (TMP and TMP dimer), and by introducing the hydrophobic residues at specific positions of mimetics to enhance the binding affinity toward the TPOR. Library was designed for peptides only, and it consisted of more than 40 peptides of all similar structures coordinates with standard amino acids only. Some of the mimetics from designed library with their binding docking score have been shown in Table 1.

**Table 1:** **Known mimetic TMP and its dimer and designed library molecular docking with TPOR results: first TMP of size 14 amino acids and TPOR were docked using the ClusPro and resulted in energy score of −798.4 kcal/mol and compared with peptides from designed library**.

Serial number	Peptide sequence	Size of peptide	Binding energy score in kcal/mol (lowest energy)	Binding energy score in kcal/mol (center)
Known mimetic (TMP)	IEGPTLRQWLAARA	14	−798.4	−703.4
TMP Dimer	IEGPTLRQWLAARA-PEG-IEGPTLRQWLAARA	28	−811.9	−685.3
**Mimetic library**				
Mim1	EDKRKSTQRRNH	12	−675.1	−616.8
Mim2	SCTYGAFDNMETYLF	15	−779.9	−660.5
Mim3	IILEYAGHYTLALA	15	−864.6	−690.6
Mim4	IILEYAGHISLEALDFQLH	19	−777.9	−697.8
Mim5	LTYAGVHVTSCTVGL	15	−834.3	−720.2
Mim6	ILEDRCTYAVLAARYG	16	−701.4	−679.7
Mim7	LLVYAGVHVTSICTEL	16	−825.2	−718.6
Mim8	EDKRKHTQRRNH	12	−678.1	−612.0
Mim9	IGLLFVLLAARAQWYL	16	−938.8	−792.5
Mim10	EDKRKHTQRRNH	12	−581.8	−507.1

*Mimetic-9 showed the high binding energy score −938.8 kcal/mol to TPOR than TMP and its dimer*.

For library designing and comparative analyses, TMP was docked with TPOR. TPOR receptor was uploaded, and its unstructured terminal residues were removed, and then TMP was uploaded as a ligand. ClusPro runs PIPER rigid body docking program and rotates the mimetic ligand with 70,000 rotations at each grid point in *x, y, z*-axis about the TPOR receptor 3D grid with spacing 1.0 Å. Then using the clustering techniques to find near-native conformations along with eliminating the non-native clusters, the 1000 best energy conformations with the lowest score were clustered and among them, 30 largest clusters were refined by minimizing the Charmm energy of the complexes. The clustering of the poses starts with the lowest energy pose and grouping all poses within 9 Å.

ClusPro resulted in a binding score (lowest energy) of −798.4 kcal/mol and center binding energy score of −703.4 kcal/mol for TMP. TMP-TPOR molecular interactions were also identified using the Dimplot and it showed; TMP interacts with TPOR with a total of fourteen hydrophobic interactions and forms seven hydrogen bonds. TMP binds to TPOR at three regions: first region (147–174) involving residues Gly147, Pro148, Pro151, Lys152, Thr155, Gly156, Pro157, Pro170, Arg174, second region (220–277) involving residues Pro223, Tyr227, Gln265, Phe267, and third region involving Phe324 of TPOR. Hydrogen bonds were found in between Leu6 of TMP and Gln173 of TPOR with 2.87 Å bond length, Arg13 of TMP and Gly220 of TPOR with 2.89 Å bond length, Ala14 of TMP and Leu221 of TPOR with 2.80 Å bond length, Ala14 of TMP and Gln222 of TPOR with 2.91 Å bond length, Gln8 of TMP and Gly263 of TPOR with 2.75 Å bond length, Gln8 of TMP and Leu264 of TPOR with 3.08 Å bond length and Pro4 of TMP and Gln277 of TPOR with 3.31 Å bond length. Furthermore, TMP dimer peptide was also docked with TPOR, and it resulted in a lowest binding score of −811.9 kcal/mol and center binding score of −685.3 kcal/mol.

**Figure 2:**
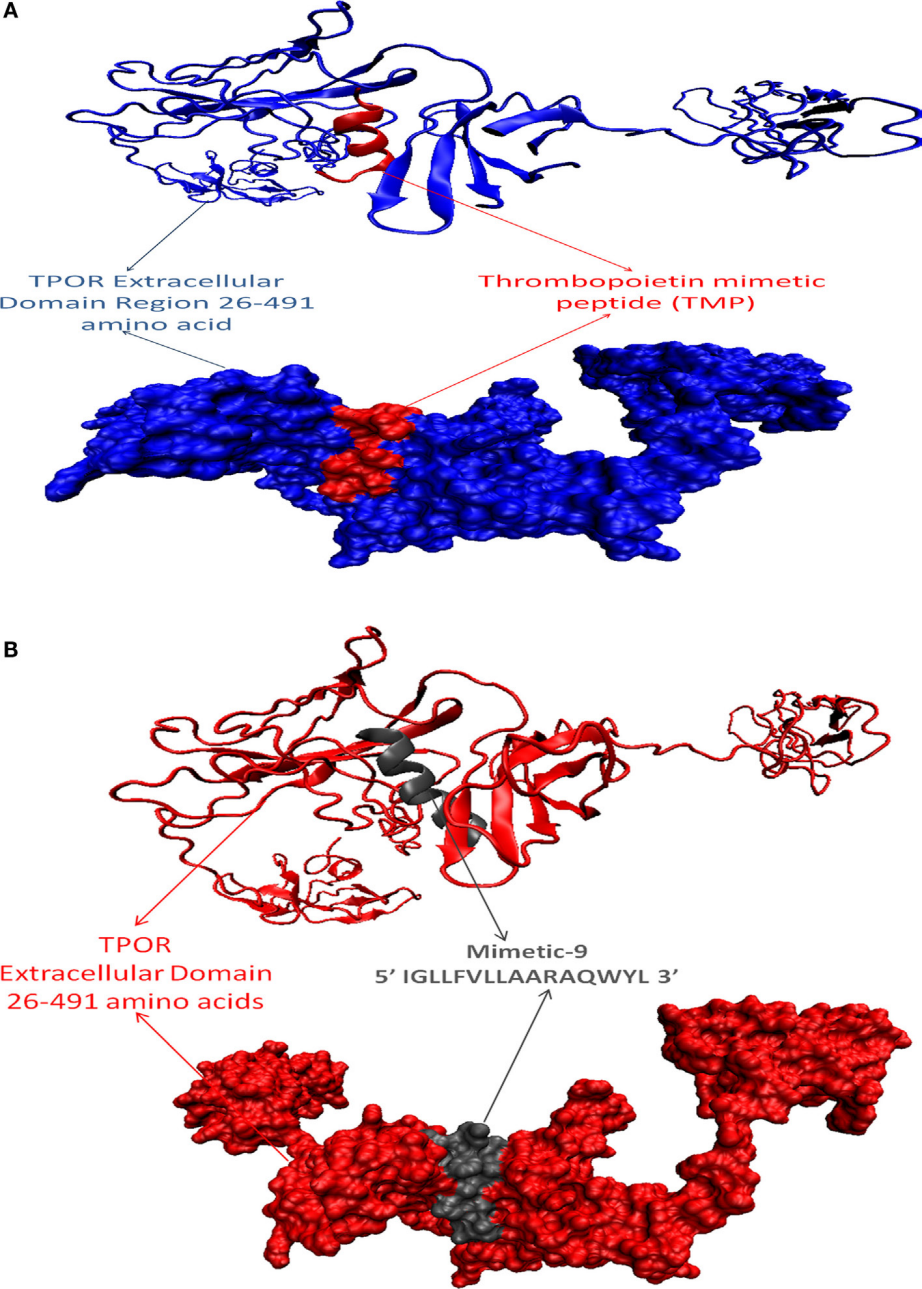
**Docked protein complexes of TMP-TPOR and mimetic-9-TPOR, in new cartoon view above and surf view below**. **(A)** TPOR extracellular domain structure of size 466 (26–491) amino acids (blue color) interacting with known mimetic TMP (red color), **(B)** TPOR extracellular domain structure of size 466 amino acids (red color) interacting with mimetic-9 (IGLLFVLLAARAQWYL) of size 16 amino acids (gray color).

**Figure 3:**
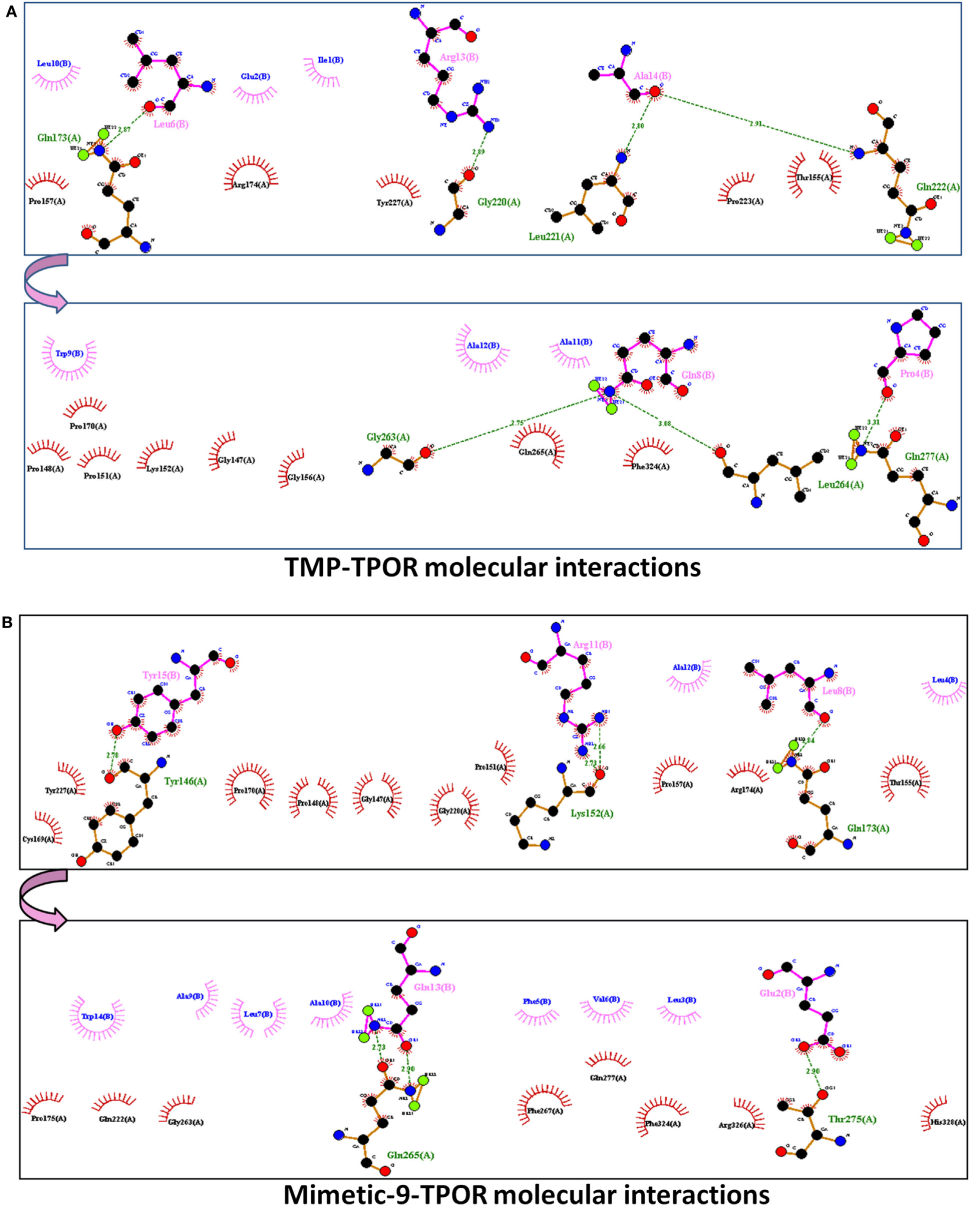
**The molecular interactions of docked protein complexes, where TPOR residues are shown in maroon color, TMP, and Mimetic-9 residues are shown in pink color**. Hydrogen bonds are shown in the green colored dashed lines and hydrophobic interaction in comb arcs. **(A)** TMP interacts with TPOR mainly at three regions at 147–174, 220–277, and 324 amino acids with seven hydrogen bonds at 173, 220, 221, 263, 264, and 277 residues of TPOR, and 14 hydrophobic interactions. **(B)** Mimetic-9 binds to TPOR more efficiently at regions 146–175, 220–277, and 324–328 amino acids with a total of seven hydrogen bonds at 146, 152, 173, 265, 275 residues of TPOR and 18 hydrophobic interactions.

After that, mimetic library was docked with TPOR using ClusPro, and mimetic-9 was found to have high-rank conformation with a weighted lowest energy score of −938.8 kcal/mol and center binding score of −792.5 kcal/mol (Figure 2). Molecular interactions involved in mimetic-9 binding to TPOR was determined which showed, mimetic-9 also binds to three regions on TPOR with more residues (18) involved in hydrophobic interactions and seven hydrogen bonds. Mimetic-9 binding regions at TPOR are: first region (146–175) with involved residues Gly147, Pro148, Pro151, Thr155, Pro157, Cys169, Pro170, Arg174, Pro175, second region (220–277) with involved residues Gly220, Gln222, Tyr227, Gly263, Phe267, Gln277, and third region (324–328) with involved residues Phe324, Arg326, and His328 (Figure 3). Hydrogen bonds were found between Leu8 of mimetic and Gln173 of TPOR with 2.84 Å bond length, Arg11 (amino position NH2) of mimetic with Lys152 (oxygen atom) of TPOR with 2.73 Å bond length, Arg11 (amine position NH1) of mimetic with Lys152 (oxygen atom) of TPOR with 2.66 Å bond length, Tyr15 of mimetic with Tyr146 of TPOR with 2.78 Å bond length, Gln13 (OE1 position) of mimetic with Gln265 (NE2 position) of TPOR with 2.90 Å bond length, Gln13 (NE2) of mimetic with Gln265 (CD position) of TPOR with 2.73 Å bond length, and Glu2 of mimetic with Thr275 of TPOR with 2.90 Å bond length. Moreover, Ala12, Leu8, Leu4, Trp14, Ala9, Leu7, Ala10, Phe5, Val6, and Leu3 residues of mimetic-9 were found to be involved in hydrophobic interactions with TPOR. Molecular docking studies clearly indicated that mimetic-9 binds more potently with TPOR than TMP and TMP dimer. And molecular interactions analyses showed that mimetic-9 binds at similar region to TPOR as TMP binds but mimetic-9 binds with more strong hydrophobic interactions and with more number of amino acid residues of TPOR, indicates the possible reason of potent binding of mimetic-9 with TPOR than TMP. Docked complex of TPOR-mimetic-9 was further analyzed with molecular dynamics simulations study and for physicochemical properties.

### Molecular Dynamics Simulation of Mimetic-9 with TPOR

**Figure 4:**
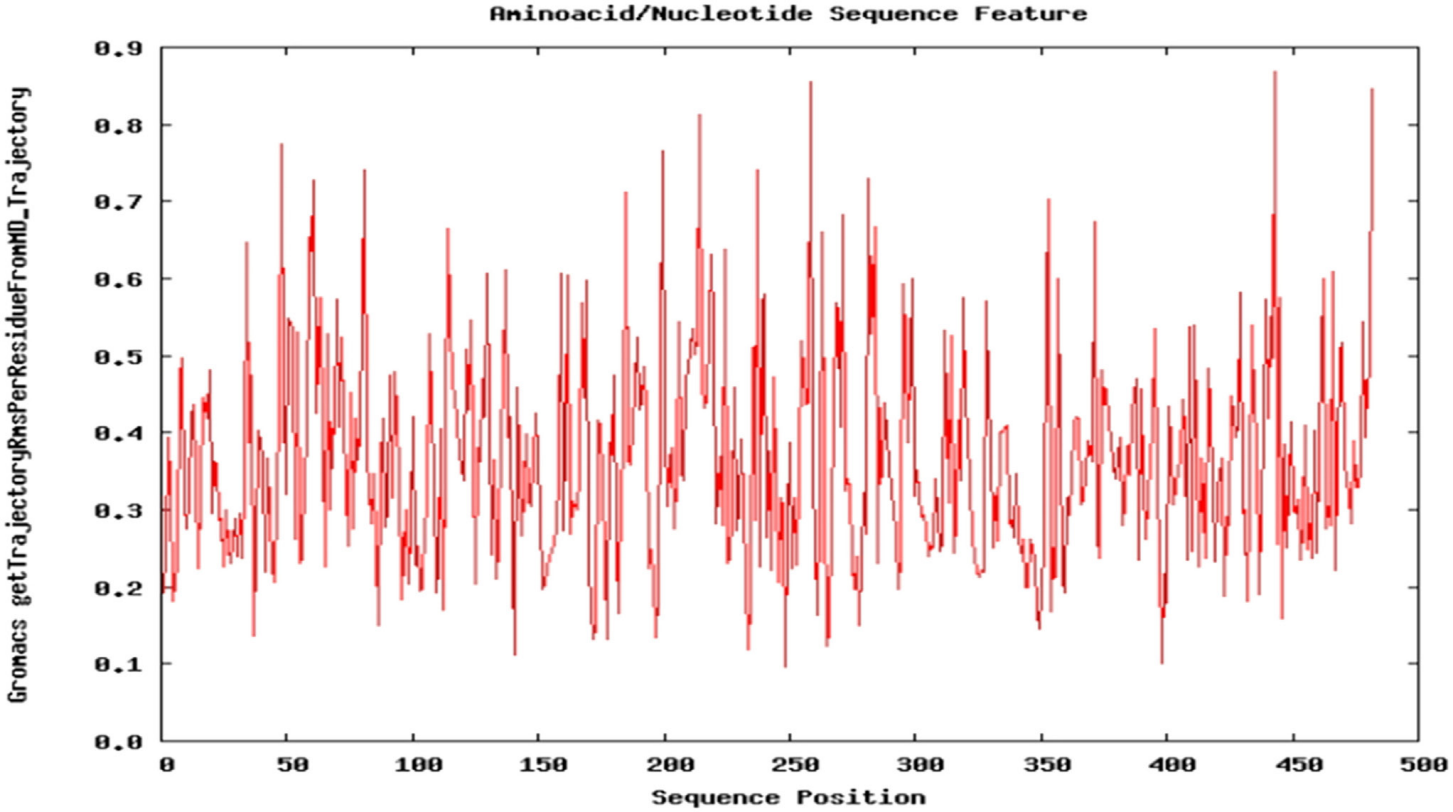
**Molecular dynamics simulation plot of the TPOR–mimetic-9 complex**. The plot depicts the structural deviation per residue, and RMSD value was found to be 0.091 Å, which suggests minimum deviation and stability of the complex.

Dynamic nature of TPOR–mimetic-9 interactions was studied through the molecular dynamics simulation for 10 ns. During the simulation, the frames of structural deviations were captured of the complex. TPOR–mimetic-9 complex ran for three frames and showed the stable conformations of interactions. Next, RMSD value was calculated and found to be 0.091 Å, which suggests the less deviation per residue of the interaction complex and stability till the end of the simulation (Figure 4).

### Physicochemical Properties and Biological Activity Assessment of Mimetic-9

Lead mimetic-9 was assessed for the physicochemical properties and compared with TMP properties (Table 2). TMP is of size 14 amino acids and its molecular weight is 1581.8 g/mol, and extinction coefficient value is 5500 M^−1^cm^−1^. TMP was found to be of basic nature with pI 9.60, and an aliphatic index value of 105 and GRAVY score of −0.150 showed that it has a comparable region of aliphatic nature and hydrophilic contents. Moreover its instability index value of 77.0, suggests it to be an unstable protein.

**Table 2:** **Physicochemical properties of lead mimetic-9 and its comparison with physicochemical properties of TMP**.

Parameters	TMP	Mimetic-9
Mimetic size	14 amino acids	16 amino acids
Molecular weight	1581.8 g/mol	1847.2 g/mol
Formula	C_71_H_116_N_22_O_19_	C_92_H_143_N_21_O_19_
Isoelectric point	9.60	8.75
Nature	Basic	Basic
Overall charge	Positive (+)	Positive (+)
Aliphatic index value	105	183.12
Instability value	77.0 (unstable)	9.38 (Stable)
GRAVY	−0.150	1.581
Extinction coefficients	5500 M^−1^ cm^−1^	6990 M^−1^ cm^−1^

Lead mimetic-9 is of size 16 amino acids and has molecular weight 1847.2 g/mol and an pI 8.75, which showed that it has a small molecular weight and basic nature. Mimetic-9 possesses the higher extinction coefficient value of 6990 M^−1^cm^−1^ indicating the presence of Tyr, Trp, and Cys in abundance. Extinction coefficient value illustrates the absorbance of light by mimetic and help in quantifying the formation and concentration of peptides. The aliphatic index value of 183.12 showed volume occupied by aliphatic side chains and GRAVY value of 1.581 suggests more volume of the mimetic-9 structure is hydrophobic. These properties aid to the isolation, physical separation, and other quantitative protein interactions studies of the mimetic. Importantly, mimetic-9 was found to be stable with instability value of 9.38. Mimetic-9 was also assessed for its subcellular localization in the eukaryotes in the physiological environment using Cello prediction server. Cello prediction server showed mimetic-9 localization in mitochondrial, nuclear organelle, and into the extracellular region with a high-reliability score.

## Discussion

The cytokine receptor TPOR recognized, which is regulated by its ligand TPO to regulate the differentiation and maturation of MKs to form platelets; TPO has been widely used to developing the methods for large-scale production of platelets, which could be used for the various clinical regimen. As discussed in the Section “Introduction,” researchers have used various strategies to develop different compounds mimicking TPO activities including peptides and chemical molecules. Present studies indicate the designing of a novel TPO mimetic that could mimic TPO role and bind TPOR potently. This molecule has the potential to trigger a signaling cascade that leads to the proliferation and differentiation of MKs into platelets.

The 3D structure of TPOR ECD region was generated through *de novo* method using I-TASSER. TPOR structure was validated with its stereochemical properties, physicochemical properties, and other structural assessment properties. Ramachandran plot of TPOR structure showed that 81% residues of the structure were in the allowed region and also possess good value for the solvation energy, torsion angle energy, solvent accessibility, and atom pairwise energy of the structure conformations.

Next, a library of peptide mimetics was designed on the basis of TPO and TMP binding sites to the TPOR and their analogs. The mimetic library constructed by us was analyzed for their binding efficiency with TPOR using molecular docking and molecular dynamic simulations. Through molecular docking analyses, we first studied the TMP interaction with TPOR, and it showed the binding energy score of −798.4 kcal/mol with seven hydrogen bonds and 14 residues involved in the hydrophobic interactions. TMP binds to TPOR majorly at three regions at 147–174, 220–277, and 324 amino acids. Moreover, TMP dimer was analyzed for its interaction with TPOR, which showed binding lowest energy score of −811.9 kcal/mol and binding energy score of −685.3 kcal/mol at center.

Then, constructed library was assessed for their interaction with TPOR. Among all molecules, mimetic-9 was found to bind most potently with highest binding energy score of −938.8 kcal/mol. Mimetic-9 was also found to interact with TPOR at three regions 146–175, 220–277, and 324–328 amino acid positions, which are similar regions where TMP interacts with TPOR but mimetic-9 interacts with more molecular interactions. Mimetic-9 forms 7 hydrogen bonds with TPOR, and 18 residues of TPOR were involved in strong hydrophobic interactions with the mimetic-9 (Table 1). Mimetic-9 was found to have strong hydrophobic interactions with more number of residues of TPOR, which showed more efficient binding of mimetic-9 with TPOR at the similar binding region as TMP.

Furthermore, the mimetic-9-TPOR complex was evaluated using molecular dynamic simulation to study its dynamic nature and stability of the complex. Molecular dynamic simulation analysis resulted in three frames during the run of simulation for 10 ns, and RMSD value was found to be 0.091 Å, which suggests the stable conformation of an interacting complex of mimetic-9 and TPOR. The physicochemical properties analysis showed that mimetic-9 is of fundamental nature with aliphatic inner side chain region, which indicates its thermostability at a wide range of temperature. GRAVY value represents the hydrophilicity and hydrophobicity characteristics of the mimetic through a scale formed on the basis of the experimental literature of hydropathy values of each amino acids side chains. The positive GRAVY score of mimetic-9 indicates its potential hydrophobic residue content.

*In vitro* stability of TMP and mimetic-9 was also analyzed by determining the instability index value. Instability index value estimated that TMP is unstable *in vitro*, and importantly mimetic-9 was found to be a stable protein from statistical analysis of experimentally defined stable and unstable proteins. Also, mimetic-9 was found to be majorly localized in the extracellular region, and since TPOR is a surface receptor, mimetic-9 at extracellular region can bind more efficiently, which is another suitable characteristic of the mimetic.

## Conclusion

In the present work, a novel TPO mimetic was designed, and it was compared with previously reported TMP for interaction with TPOR using the molecular docking simulation analyses and was found to be more potent than TMP. Novel Mimetic-9-TPOR complex showed a stable binding with an RMSD value of 0.091 Å through molecular dynamics simulation. Furthermore, physicochemical properties of mimetic-9 showed that it is a stable protein with high instability index value and good GRAVY score. Moreover, it is predominantly localized to an extracellular region, a prerequisite property for mimetic to interact with cell surface receptor TPOR.

## Author Contributions

Methods and experimental setup were designed by NK, RC and VKS and methods were implemented by NK, MK and AS. Manuscript was written by NK and MK and revised by VKS. The final manuscript has been read and approved by all the authors.

## Conflict of Interest Statement

The authors declare that the research was conducted in the absence of any commercial or financial relationships that could be construed as a potential conflict of interest.

## Footnotes

^1^http://blast.ncbi.nlm.nih.gov/Blast.cgi

^2^http://zhanglab.ccmb.med.umich.edu/I-TASSER/

^3^http://swissmodel.expasy.org/qmean/cgi/index.cgi

^4^http://mordred.bioc.cam.ac.uk/~rapper/rampage.php

^5^http://bioserv.rpbs.univ-paris-diderot.fr/services/PEP-FOLD3/

^6^http://web.expasy.org/protparam/

^7^http://cello.life.nctu.edu.tw/
